# A Cost Consequence Analysis of Seven Diagnostic Strategies for Ovarian Cancer: A Model‐Based Economic Evaluation

**DOI:** 10.1111/1471-0528.70074

**Published:** 2025-11-05

**Authors:** Samuel J. Perry, Donal Griffin, Eleanor V. Williams, Tracy E. Roberts, Fong Lien Kwong, Sarah Williams, Jon Deeks, Katie Scandrett, Ridhi Agarwal, Sudha S. Sundar, Mark Monahan

**Affiliations:** ^1^ Department of Applied Health Sciences, College of Medicine and Health University of Birmingham Birmingham UK; ^2^ Department of Cancer and Genomic Sciences University of Birmingham Birmingham UK; ^3^ University Hospitals Birmingham Birmingham UK; ^4^ NIHR Birmingham Biomedical Research Centre, University Hospitals Birmingham NHS Foundation Trust University of Birmingham Birmingham UK; ^5^ Pan Birmingham Gynaecological Cancer Centre Midlands Metropolitan University Hospital Birmingham UK

**Keywords:** cost‐effectiveness, economic evaluation, gynaecologic cancer, menopause, ultrasound

## Abstract

**Objective:**

To assess the costs and consequences of seven diagnostic strategies for ovarian cancer in pre‐ and post‐menopausal women with symptoms in secondary care.

**Design:**

Economic evaluation alongside a prospective single‐arm diagnostic accuracy study.

**Setting:**

NHS secondary care outpatients (2‐week referrals, clinics, GP referrals, cross‐specialty referrals) and inpatients (emergency presentations to secondary care).

**Sample:**

Two cohorts of 857 pre‐menopausal and 1242 post‐menopausal women newly presenting to secondary care with symptoms of suspected ovarian cancer.

**Methods:**

A model‐based cost‐consequence analysis (CCA) was conducted using a decision tree simulating patient pathways over 12 months. Diagnostic accuracy data were sourced from the ROCkeTS study and supplemented by literature.

**Main Outcome Measures:**

Cancer deaths, correct diagnosis proportion, and diagnostic yield.

**Results:**

No diagnostic strategy was optimal across all outcomes. Across both cohorts, the Risk of Malignancy Index (RMI) 200 was least expensive but had poor cancer death and diagnostic yield outcomes. The ADNEX 3% strategy had the highest diagnostic yield and lowest cancer mortality but was the most expensive. For pre‐menopausal women, the IOTA ADNEX 10% strategy outperformed ORADS, ROMA, and CA125 in cost and outcomes. For post‐menopausal women, the high cancer prevalence required a trade‐off. In sensitivity analysis, a two‐step IOTA ADNEX 10% strategy outperformed ORADS, ROMA, and CA125 across all three outcomes, making the strategy a more balanced choice in both cohorts.

**Conclusion:**

At 12 months, no single diagnostic strategy was superior. Early diagnosis requires balancing cancer mortality, diagnostic yield, and cost. The IOTA ADNEX two‐step strategy at a 10% threshold provided the best trade‐off across these factors and is recommended for practice.

## 1 Introduction

Ovarian cancer remains a significant health concern worldwide, with over 300 000 new cases diagnosed annually [1]. In the UK, approximately 7500 new cases are diagnosed each year [2], with around 70% diagnosed at advanced stages [3] necessitating extensive treatment including maximum cytoreductive surgery, chemotherapy, and targeted therapy [4]. Due to the high proportion of late‐stage diagnoses, ovarian cancer has a substantial mortality burden, with approximately 4100 deaths occurring annually in the UK [2]. Whilst most cases occur post‐menopause, some pre‐menopausal women are diagnosed each year [5].

Women presenting to general practitioners in the UK are tested with cancer antigen 125 (CA125) and ultrasound and referred to secondary care if these are abnormal [4]. However, adherence to this guidance varies in clinical practice [6]. In hospital, women receive a triage test to assess their risk of ovarian cancer. Professional societies endorse different risk prediction models for ovarian cancer. The Risk of Malignancy Index (RMI) is the UK standard of care [4], whilst IOTA ADNEX [7], ORADS [8] and Risk of Ovarian Malignancy Algorithm (ROMA) [9] are used internationally. Effective triage is critical to ensure high‐risk patients receive appropriate surgery with the best outcomes [10].

Current UK guidance endorses the RMI 200–250 strategy, which has 70% sensitivity and 90% specificity [11, 12]. Thresholds of 200 or 250 are widely used in clinical practice, and NICE guidance (CG122) specifically supports the use of an RMI threshold of 250 to identify individuals at high risk of underlying ovarian cancer [4]. To compensate for poor sensitivity, surgery is recommended irrespective of diagnostic outcome: women with RMI ≥ 200 undergo surgery at a designated gynaecological cancer centre by a specialist gynaecological surgical oncologist, whereas women with RMI < 200 undergo surgery at a cancer unit by a gynaecologist. This disadvantages women with early‐stage cancer who undergo surgery by non‐cancer specialists and those without cancer who may have avoided unnecessary surgery with a more accurate test. The high rate of surgical intervention under the current pathway (70% in the ROCkeTS study) [14] demonstrates the need for refined tools to improve cancer detection while minimising unnecessary surgeries and associated morbidity.

The Refining Ovarian Cancer Test Accuracy Scores (ROCkeTS) study externally validated risk prediction models estimating the probability of ovarian cancer for women with non‐specific symptoms in pre‐ and post‐menopausal cohorts [13]. ROCkeTS was a prospective single‐arm diagnostic accuracy study where all patients received all commonly used tests and diagnostic accuracy was evaluated against gold standard histology or 12‐month outcome [13]. The test accuracy results are published elsewhere [14]. Here, we report the economic evaluation conducted alongside ROCkeTS, assessing the costs and consequences of seven different ovarian cancer diagnostic testing strategies in two cohorts (pre‐ and post‐menopausal women) using ROCkeTS data.

## 2 Methods

### 2.1 Study Design of ROCkeTS Prospective Study

Participants were eligible for inclusion in the ROCkeTS study if they were aged 16–90 years and newly presenting to secondary care with symptoms and either an abnormal CA125 test, abnormal imaging, or both. Participants donated a sample of blood and underwent a transvaginal and transabdominal ultrasound performed mainly by NHS sonographers with certified training in the use of IOTA ultrasound models [13]. The study recruited women at ‘low’ or unknown genetic risk of ovarian cancer, that is, women not known to have a BRCA or other germline mutation predisposing to ovarian cancer. Menopause was defined as more than 12 months without menstruation. Women who had not menstruated for more than 12 months due to contraception or hysterectomy were categorised by age: those under 50 as pre‐menopausal and those 51 and over as post‐menopausal. Women who were still menstruating and over 50 years old were considered pre‐menopausal. The ovarian cancer definition used in this study is the WHO definition which is a group that includes primary malignancies of the ovary, fallopian tube, and primary peritoneal cavity [15].

### 2.2 Current Standard of Care Diagnostic Pathway

When a patient presents with symptoms of ovarian cancer in secondary care, usual practice involves a series of diagnostic tests and procedures. The RMI I score is calculated which combines CA125, limited ultrasound variables, and menopausal status. Women with an RMI I score ≥ 200 are referred to a cancer specialist multidisciplinary team and a CT scan of the thorax as well as the pelvis and abdomen as part of the diagnosis work‐up for suspected ovarian cancer [4].

Some women will have an image‐guided biopsy, and others will undergo primary surgery to obtain a histological diagnosis [11, 12]. Patients with RMI 25–200 undergo surgery in a cancer unit and may also undergo an MRI scan. Those considered at low risk of cancer (RMI < 25) will be managed under surveillance with follow‐up scans [12].

### 2.3 Cost Consequence Modelling Methods

A model‐based cost‐consequence analysis (CCA) was undertaken using a decision tree developed in TreeAge Pro 2024 [16]. A UK National Health Service (NHS) perspective was adopted [17]. The model structure and methods followed the Consolidated Health Economic Evaluation Reporting Standards (CHEERS) checklist [18] (Table S1). The modelled diagnostic strategies considered where a patient was identified as having ovarian cancer were as follows and illustrated in schematic Figure S1 in the appendix:

- RMI I test score ≥ 200
  ○ RMI is a scoring system used to estimate the likelihood that an ovarian mass is malignant. A score ≥ 200 indicates a high risk of ovarian malignancy [19]. A score of ≥ 200 was chosen since RCOG guidance for postmenopausal women refers to both thresholds of ≥ 200 and ≥ 250, with no explicit preference between them, whilst premenopausal guidance does not advocate for a specific threshold [11, 12]. Additionally, previously published analysis from the ROCkeTS study found no statistically significant difference in performance of RMI at 200 or 250 thresholds [14, 20].
- ROMA test result of manufacturer recommended thresholds (> 29.9% for post‐menopausal cohort; > 11.4% for pre‐menopausal cohort).
  ○ ROMA uses blood markers CA125 and HE4 (human epididymis protein 4) along with menopausal status to estimate cancer risk [21]. Results above the thresholds indicate a high risk of epithelial ovarian cancer [9].
- IOTA ADNEX test result
  ○ IOTA ADNEX model is used to differentiate between benign and malignant ovarian tumours [22]. The model uses seven ultrasound variables and three clinical parameters (age, optional CA125 level and setting) [23]. Two different thresholds are considered, at 10% and at 3%.
  ○ CA125 test result ≥ 35 u/mL
- CA125 is a blood test measuring a protein linked to ovarian cancer. A level above 35 u/ml is considered elevated [24].
- Ovarian‐ADNEXal Reporting and Data System (ORADS) test result ≥ 4
  ○ ORADS is a standardised imaging classification for ovarian masses based on pattern recognition in ultrasound. A score ≥ 4 means there is a moderate‐to‐high suspicion of malignancy (> 10%) [8].

The outcomes considered in the CCA were cancer death, proportion of correct diagnosis and diagnostic yield. A correct diagnosis was where an individual with ovarian cancer was correctly identified as being at high risk of the disease (true positive) or where an individual without ovarian cancer was correctly identified as being at low risk of the disease (true negative). An incorrect diagnosis was where an individual without ovarian cancer was incorrectly identified as being at high risk of having the disease (false positive) or where an individual with ovarian cancer was incorrectly identified as being at low risk of the disease (false negative). Diagnostic yield is the percentage of cancers detected out of the population screened. These outcome measures were chosen to provide a balanced assessment of diagnostic strategy performance. Diagnostic yield quantifies the ability to detect cancer cases within the population, favouring strategies with higher sensitivity. Proportion of correct diagnosis incorporates both sensitivity and specificity to evaluate overall diagnostic accuracy and, combined with prevalence data, quantifies false positive and false negative rates across patient cohorts. Cancer deaths were chosen as a patient‐centred outcome of key interest to policymakers, inherently favouring strategies with higher sensitivity given the critical importance of early cancer detection for survival outcomes. The pathways were the same for both cohorts and differed only by the test characteristics and underlying ovarian cancer prevalence in each population. Model inputs are listed in Tables S2–S4.

A 12‐month end point was used as the time horizon for this model. This was chosen for model feasibility reasons to comprehensively map out the different pathways without making the decision tree unmanageably complex by looking beyond the 12 months in a comprehensive manner. We felt that this was preferable to the alternative of looking beyond the 12 months at the expense of much‐simplified pathways that do not encapsulate the nuance and complexities of longer‐term treatment management pathways. Discounting was not undertaken due to the time horizon length. A cost year of 2022–2023 was used for the model. Costs were inflated where applicable using the PSSRU inflation index [25].

It was decided a CCA best represented the different outcome measures and trade‐offs associated with diagnosis. When considering alternative approaches such as a cost‐effectiveness analysis we found no one outcome measure would sufficiently represent patients with and without ovarian cancer. Furthermore, the decision against cost‐utility analysis (CUA) was due to ROCkeTS not measuring health‐related quality of life (HRQoL) data as this was a diagnostic test accuracy study. We also found insufficient utility data available from previous studies and databases to accurately reflect all outcomes when using a comprehensive patient pathway mapping approach.

### 2.4 Model Assumptions

Several simplifying assumptions were required to develop a workable model given data limitations and variations in care recommendations. These were agreed upon by the clinical research team prior to conducting the analysis.

All ovarian cancer patients were assumed to receive treatment, meaning best supportive care was not an option. All patients were assumed to respond to first‐line platinum‐based chemotherapy; therefore second‐line treatment was not considered within the 12‐month timeline. First‐line treatment was assumed to take 6 months from test result, and maintenance therapy (FIGO Stage II‐IV, varied by BRCA/HRD status) was for the remaining 6 months. Patients identified as BRCA germline mutation carriers or HRD‐positive were considered equivalent and eligible for Olaparib PARP inhibitor maintenance therapy, where appropriate. Patients who were BRCA‐negative and HRD‐negative were assumed to receive Niraparib PARP inhibitor maintenance therapy, where appropriate. Adjuvant therapy, including bevacizumab for eligible advanced‐stage patients, was incorporated uniformly across diagnostic strategies, with details with cost inputs provided in Supporting Information.

Treatment or tumour testing was not differentiated by ovarian cancer histology types. All chemotherapy patients had a computed tomography (CT) scan before the first cycle, and after the third and sixth cycles. All follow‐ups up to 12 months consisted of CA125 tests and CT scans with a gynaecologist in secondary care. Stage‐specific ovarian cancer deaths were taken from NHS Digital and comprise the 12‐month age‐standardised ovarian cancer deaths of adult patient ovarian cancers diagnosed from 2016 to 2020 in England [26].

Key assumptions are provided in Table 1 for the TP, TN, FP, and FN patient pathways, with further details provided in Supporting Information (18–21). Figures S2–S8 illustrate patient movement across pathways for each diagnostic category.

**TABLE 1:** Key test result patient pathway assumptions.

Pathway	Key assumptions
True positive	Stage I: Primary surgery alone, resulting in no residual disease
	Stage II‐III: Primary surgery with adjuvant carboplatin plus paclitaxel (CarboTaxol) chemotherapy, resulting in no residual disease
	Stage IV: Delayed debulking surgery with CarboTaxol plus bevacizumab chemotherapy
	Stage II‐IV: Maintenance chemotherapy – Olaparib for BRCA/HRD positive, Niraparib for BRCA/HRD negative
False negative	Either immediate surgery in secondary care for symptoms/suspected benign histology, cancer identified from histology
	Or cancer correctly identified at 6‐month follow‐up
	23% of Stage I patients are upstaged to Stage II‐III [24]
	Modifications to stage‐specific treatment compared to TP are detailed in Supporting Information (18–21)
False positive	Initial management either through staging CT alone or staging CT plus MRI
	Staging CT alone assumed to suggest Stage I cancer, undergoing staging laparotomy with histology indicating absence of cancer
	Staging CT plus MRI sufficient to rule out cancer
True negative	Either managed surgically for symptoms/suspected benign histology
	Or discharged with two 6‐month follow‐up gynaecologist consultations in secondary care

*Note:* See Supporting Information 18–21 for full details of assumptions.

### 2.5 Analysis

The analysis was stratified by the pre‐ and post‐menopausal cohorts due to a higher ovarian cancer prevalence amongst post‐menopausal women, and the potential adverse fertility impact of surgical intervention on pre‐menopausal women [5]. Strategies were ranked by costs, and an incremental approach was taken whereby strategies were compared against the nearest alternative in cost. A strategy was considered dominated if it was more expensive and less effective on all outcome measures compared to a strategy.

### 2.6 Deterministic Sensitivity Analysis

The following deterministic sensitivity analyses (DSAs) were conducted to assess the impact on the base case results for both cohorts:

1. The prevalence of ovarian cancer was varied ±25%.
2. The lower and upper confidence intervals of the upstaging variable from Van de Vorst [27].
3. The proportion of FPs that have additional MRI was varied ±25%.
4. Proportion of TPs with Stage I Ovarian Cancer was varied ±25%.
5. Proportion of correctly staged resectable patients who have all listed surgery procedures was varied ±25%.

We also conducted the analysis using the alternative threshold value of ≥ 250 for the RMI I test score, since guidance for the management of ovarian masses in postmenopausal women refers to both ≥ 200 and ≥ 250 thresholds [11]:

6 RMI I test score threshold was increased to ≥ 250.

Lastly, the ROCkeTS study assessed the performance of ultrasound models when used by NHS sonographers with varying levels of expertise, ensuring they had received appropriate training, certification and quality assurance. To objectively evaluate these models, the ROCkeTS diagnostic accuracy analysis excluded subjective assessments of ovarian masses during ultrasound examinations. This approach isolated the performance of ultrasound‐based models and minimized bias linked to individual operator expertise. In clinical practice, an alternative approach could triage out approximately 36% of women with benign‐appearing tumors during the same ultrasound examination using a two‐step strategy [28]. To account for this, we conducted a final DSA:

7 For the ADNEX 10% strategy, the sensitivity and specificity of a two‐step strategy was used [28] with an absolute reduction of 17% in surgeries for FPs [29, 30]

### 2.7 Probabilistic Sensitivity Analysis

Probabilistic sensitivity analysis (PSA) was conducted to explore model input uncertainty. The distributions and parameters used are shown in Tables S3 and S4. Beta distributions were attached to the test proportions and count data. No distribution was attached where there were zero count data. Gamma distributions were attached to cost data, with a 20% standard error assumed where information was unavailable. 10 000 iterations were run with a seeded random generator. Mean costs and outcomes were calculated across the iterations along with their 95% prediction intervals. Incremental cost‐consequences were presented to illustrate parameter uncertainty with separate scatterplots for each outcome measure A 95% confidence ellipse is also presented within the incremental cost‐ consequence scatterplot to convey the region where 95% of the incremental iterations resided [31].

## 3 Results

The base case deterministic results for the pre‐menopausal cohort (*n* = 857) and post‐menopausal cohorts (*n* = 1242) are shown in Table 2. In the pre‐menopausal cohort, 5.7% were diagnosed with ovarian cancer, 87.5% had no ovarian cancer, and 6.8% fell into other categories. In the post‐menopausal cohort 17.3% were diagnosed with ovarian cancer, 69.3% had no ovarian cancer and 13.4% were classified as other.

**TABLE 2:** Base case deterministic results.

Strategy	Cost (£)	Ovarian cancer deaths (%)	Proportion of correct diagnosis (%)	Diagnostic yield (%)
*Pre*				
RMI 200	5283	1.17	92.51	3.00
ADNEX 10%	6823	1.06	75.99	5.47
ORADS	6964	1.12	84.59	4.86
CA125	7609	1.43	65.15	4.76
ADNEX 3%	7795	1.05	48.47	5.73
ROMA 11.4%	8056	1.13	73.45	4.86
*Post*				
RMI 200	13 471	3.84	84.52	16.94
ORADS	14 243	4.02	77.92	15.22
CA125	14 414	4.04	79.27	17.14
ADNEX 10%	14 471	3.73	65.99	19.15
ROMA 29.9%	14 494	3.96	81.51	17.53
ADNEX 3%	15 119	3.70	44.59	19.93

For both cohorts, the RMI 200 strategy was the least costly and had the highest proportion of correct diagnoses but performed poorly on diagnostic yield relative to other strategies. The ADNEX 3% strategy was associated with the lowest number of cancer deaths and highest diagnostic yield in both cohorts. For the pre‐menopausal cohort, the ORADS, ROMA 11.4% and CA125 strategies were dominated by the ADNEX 10% strategy. Given the high prevalence of ovarian cancer in post‐menopausal women, selecting a diagnostic strategy involves a trade‐off. Costs were higher in the post‐menopausal cohort compared to the pre‐menopausal cohort, as there is a higher cost associated with cancer cases, the increased prevalence of ovarian cancer has contributed to the higher overall per patient cost in the post‐menopausal cohort compared to the pre‐menopausal cohort.

The DSA results are shown in Tables S5 and S6. Decreasing the ovarian cancer prevalence improved the proportion of correct diagnoses for strategies with higher specificity such as RMI 200 while increasing ovarian cancer prevalence improved the proportion of correct diagnoses for strategies with higher sensitivity such as ADNEX 3%. Changing the ovarian cancer prevalence had the greatest impact on the expected cost per strategy, although the order of strategies by cost remained consistent with the base case analysis. Increasing the RMI threshold to 250 had no impact on the findings.

For the two‐step ADNEX 10% strategy the expected costs and diagnostic yield decreased while cancer deaths and the proportion of correct diagnoses increased relative to baseline in both cohorts. In the post‐menopausal cohort, this reduction in cost meant the two‐step ADNEX 10% category now had the second lowest cost after RMI 200, whilst it retained this ordering from the base case in the pre‐menopausal cohort. As such, for both cohorts the ORADS, ROMA, and CA125 strategies were dominated by the two‐step strategy. Furthermore, the two‐step strategy continued to outperform RMI 200 on cancer deaths and diagnostic yield, whilst the disparity in the proportion of correct diagnoses narrowed.

The mean PSA iterations for the two cohorts are shown in Tables 3 and 4 with associated 95% prediction intervals for the costs and consequences. The average costs and outcome measure effectiveness were similar to the deterministic base case results.

**TABLE 3:** PSA mean output premenopausal cohort.

Strategy	Costs (£)	Ovarian cancer death	Correct diagnosis proportion	Diagnostic yield
RMI 200	5276 (5264 to 5289)	0.01176 (0.01172 to 0.0118)	0.9252 (0.925 to 0.92539)	0.03001 (0.02989 to 0.03012)
CA125	7582 (7566 to 7598)	0.01438 (0.01433 to 0.01443)	0.73443 (0.73411 to 0.73474)	0.04847 (0.04832 to 0.04862)
ROMA 29.9%	8034 (8017 to 8052)	0.01138 (0.01134 to 0.01142)	0.48466 (0.48426 to 0.48505)	0.05725 (0.05709 to 0.05741)
ORADS	6947 (6931 to 6962)	0.01118 (0.01114 to 0.01122)	0.75985 (0.75951 to 0.76019)	0.0546 (0.05445 to 0.05476)
ADNEX 10%	6795 (6780 to 6809)	0.01062 (0.01058 to 0.01066)	0.65148 (0.65115 to 0.65181)	0.04747 (0.04732 to 0.04761)
ADNEX 3%	7759 (7742 to 7776)	0.01052 (0.01048 to 0.01056)	0.84598 (0.84573 to 0.84624)	0.04855 (0.0484 to 0.0487)

**TABLE 4:** PSA mean output postmenopausal cohort.

Strategy	Costs	Ovarian cancer death	Correct diagnosis proportion	Diagnostic yield
RMI 200	13 460 (13 436 to 13 485)	0.03842 (0.03836 to 0.03848)	0.84484 (0.84461 to 0.84507)	0.16924 (0.16902 to 0.16947)
CA125	14 417 (14 391 to 14 442)	0.04034 (0.04028 to 0.04041)	0.81502 (0.81477 to 0.81526)	0.17512 (0.17489 to 0.17535)
ROMA 29.9%	14 482 (14 456 to 14 507)	0.03957 (0.03951 to 0.03964)	0.44622 (0.44591 to 0.44654)	0.19908 (0.19884 to 0.19932)
ORADS	14 237 (14 212 to 14 262)	0.04014 (0.04008 to 0.0402)	0.65945 (0.65913 to 0.65976)	0.19136 (0.19112 to 0.1916)
ADNEX 10%	14 478 (14 452 to 14 503)	0.03728 (0.03722 to 0.03734)	0.79244 (0.7922 to 0.79268)	0.17127 (0.17104 to 0.17149)
ADNEX 3%	15 113 (15 085 to 15 140)	0.03696 (0.0369 to 0.03702)	0.7789 (0.77865 to 0.77915)	0.15221 (0.15199 to 0.15243)

The incremental cost‐consequence scatterplots for the three outcome measures for the pre‐menopausal cohort and post‐menopausal cohort are shown in Figures S9–S23 and Figures S24–S38, respectively. All scatterplots used RMI 200 as a baseline comparator.

For the pre‐menopausal cohort, the RMI 200 strategy was less expensive in the majority of the iterations relative to the other comparators. ROMA 11.4% was consistently more expensive than RMI 200 across iterations. For cancer deaths, the PSA output indicates an inverse relationship between cost and deaths for the incremental iterations. For correct diagnosis proportion, no strategy had a better correct diagnosis proportion than the RMI 200 strategy across the PSA iterations. Furthermore, for the diagnostic yield, all the other strategies performed better on diagnostic yield compared to RMI 200.

For the post‐menopausal cohort, the RMI 200 strategy was generally less expensive in the majority of iterations relative to the other comparators. While the inverse relationship between cost and deaths was seen again in the post‐menopausal cohort, the cancer death incremental iterations tended to be less dispersed compared to the pre‐menopausal cohort ones. For correct diagnosis proportion, RMI 200 had a higher correct diagnosis proportion than ORADS, ADNEX 10% and ADNEX 3%. The RMI 200 strategy had a much higher correct diagnosis proportion than ROMA 29.9% and CA125 strategies. Furthermore, as with the pre‐menopausal cohort, all the other strategies performed better on diagnostic yield compared to the RMI 200 strategy.

## 4 Discussion

### 4.1 Principal Findings

This study assessed the costs and consequences of seven diagnostic strategies in ovarian cancer screening. Decision tree modelling comprehensively mapped the patient pathway for pre‐ and post‐menopausal women with suspected ovarian cancer, capturing key costs and clinical consequences during the first 12 months after diagnostic testing. No single strategy outperformed all others across all outcome measures: cost, diagnostic yield, cancer deaths and proportion correctly diagnosed. While RMI 200 was the cheapest in both cohorts, its lower sensitivity resulted in more cancer‐related deaths and a lower diagnostic yield compared to ADNEX‐based strategies. Sensitivity analysis supported the base‐case findings across both cohorts.

The two ADNEX‐based strategies modelled in the analysis using ROCkeTS data (where the risk prediction models were tested in isolation) were characterised by high sensitivity but lower specificity, resulting in potentially unnecessary costs associated with the management of false positive results. Recently, Landolfo et al. [28] evaluated a two‐step ultrasound IOTA ADNEX strategy in 4905 patients, first applying benign descriptors for initial triage, followed by the IOTA ADNEX model. At a 10% risk threshold, they reported a sensitivity of 91% and a specificity of 85%. In a sensitivity analysis, this two‐step strategy with IOTA ADNEX 10% was modelled alongside a reduction in surgery rate of 17%, extrapolated from Nunes et al. [29] findings for ultrasound‐based models. This reduced false positives and presented the best balance across all outcome measures, making it suitable for clinical implementation. However, a two‐step strategy also slightly underestimated the risk of malignancy compared to a strategy of IOTA ADNEX alone.

### 4.2 Strengths and Limitations

A key strength of this analysis is its use of prospectively collected data from the ROCkeTS study to facilitate the comparisons across numerous diagnostic strategies. ROCkeTS is the first head‐to‐head comparison prospective study published investigating all currently used diagnostic models and tests. Additionally, the analysis stratified pre‐ and post‐menopausal cohorts to reflect key differences in prevalence of cancer, performance of diagnostic models, histology types and consequences of treatment in both groups [32, 33, 34].

ROCkeTS recruited patients who were newly referred to secondary care (hospital) from urgent suspected cancer pathway referrals (2 week wait clinics), emergency presentations and elective clinic referrals. The majority of patients recruited (67%, 1751/2596) were recruited from patients presenting to hospital through the urgent suspected cancer pathway (otherwise known as fast‐track pathway). This is a timed referral pathway for patients referred from primary care to secondary care [32]. In contrast, data from the National Ovarian Cancer Audit demonstrate that 40% of patients with OC were diagnosed within 28 days of an emergency admission. These patients are more likely to have advanced stage disease, poorer performance status, clinically apparent findings (e.g., pleural effusion, ascites, omental cake) where cross‐sectional imaging and a biopsy are the most appropriate diagnostic investigations [35]. Thus, ROCkeTS is more representative of patients referred from primary care with suspicion of ovarian cancer; this is a strength of our analysis as differences in diagnostic strategies are most likely to be impactful in women with earlier stage disease.

Another key strength is the incorporation of up‐to‐date treatment modalities, including germline testing for inherited cancer, tumour testing for HRD, and appropriate treatment with targeted therapies such as Bevacizumab and PARP inhibitors. Furthermore, the present analysis is also the first to incorporate upstaging within the model and to incorporate the impact of different diagnostic strategies on surgery rates.

This study is not without limitations. Although it employed a 12‐month time horizon to comprehensively examine how a patient would transition through treatment pathways and their associated costs within the first year from diagnosis, a longer time horizon may have given a better reflection of important differences in costs and outcomes between the alternative diagnostic strategies. For instance, differences in cancer deaths would likely increase over time between strategies with varying levels of sensitivity. Additionally, within the 12‐month horizon considered, costs associated with a strategy improving early accurate ovarian cancer diagnosis were higher due to longer durations of maintenance therapies. The proportion of correct diagnosis and diagnostic yield outcome measures would remain unaffected by an extended time horizon. Data from the pragmatic trial ROCkeTS underpins this cost consequence evaluation. In ROCkeTS, only 40% of women in the postmenopausal cohort and 60% of the premenopausal cohort were diagnosed with early‐stage disease (Stages 1 and 2), representing current practice. Even at the 12‐month time horizon, ovarian cancer deaths were substantial and varied considerably by cancer stage; Stage I: 2.2%, Stages II–III: 23.2% and Stage IV: 43.7%. As such, whilst our focus is on early diagnosis, we modelled the use of the diagnostic strategies on the costs and consequences of the entire patient cohort with all stages of cancer to mimic the current patient pathway where the majority present with late‐stage disease.

An additional consideration is that the two‐step ADNEX 10% strategy has not yet been prospectively evaluated within the UK healthcare setting. Consequently, there may be limitations in the generalisability of our modelled findings to routine clinical practise. The ongoing ADVOCATE study, conducted across the East of England Cancer Alliance, will provide valuable real‐world evidence to assess the feasibility and impact of this approach on surgery rates and related outcomes in the UK context.

Furthermore, surgery rates and surgical‐related morbidity were not incorporated into the analysis. Inclusion of this outcome would have allowed comparison of potential differences in postoperative complications, and their associated costs and consequences, which were not captured in the current model. Whilst our CCA has focused on cancer mortality as a key outcome of interest to patients, clinicians and policy makers, we acknowledge that other outcomes such as surgery rates, and surgery‐related morbidity are important measures. As practice in ROCkeTS was as per standard of care strategy RMI, we did not have information on surgery rates from alternative strategies and thus we did not use surgery rates as an outcome measure. Use of alternate diagnostic strategies may reduce surgery rates. A further limitation was the inability to model the impacts of ovarian cancer on fertility and related complications in premenopausal women. Ovarian cancer treatment in premenopausal women frequently results in infertility due to the aggressive nature of the surgical procedures and the effects of chemotherapy [5]. While fertility preservation techniques exist, their applicability is constrained by factors like cancer stage and patient health [5]. Survivors of premenopausal ovarian cancer also face challenges including premature menopause, reduced ovarian reserve, and increased risk of longer‐term cardiovascular complications [36]. Additionally, in contrast to published literature, the prevalence of primary ovarian cancer in ROCkeTS was lower (17% postmenopausal cohort, 5.5% premenopausal cohort) reflecting our recruitment of patients referred from primary care through urgent suspected cancer pathways. Unfortunately, current available national data do not identify those referred specifically as urgent suspected ovarian cancer to assess how the prevalence of cancer within the ROCkeTS dataset compares with national trends—data on cancer outcomes is only available for a broader category of suspected gynecological cancer [37]. Ovarian cancer is a rare disease, and we acknowledge that prevalence in our study is likely representative of secondary care (hospital settings) but higher than would be expected in community/primary care studies where a GP would expect to see only one in 400 women with ovarian cancer. In addition, in comparison to national datasets ROCkeTS had a higher proportion of women with early‐stage cancer (stages 1 and 2) (Table S7).

While the results from this CCA are globally relevant—diagnostic work‐up, triage to gynaecological oncology care, staging and treatment guidelines are consistent with international best practice [38, 39]—we acknowledge that practice varies, particularly in countries with private health sector systems where thresholds for surgical intervention may vary, and therefore our findings may not be fully applicable.

Finally, this study did not include a CUA, limiting a full assessment of cost‐effectiveness and the impact of timely diagnosis on HRQoL and HRQoL decrements for misdiagnosed patients. However, no appropriate literature sources were identified to provide information on the disutility of screening for all types of patients with suspected ovarian cancer [40]. Previous economic evaluations conducting CUA for ovarian cancer diagnostic testing have relied on sources limited by a small sample size and health state descriptions that do not generalise to current management of suspected ovarian cancer patients [4, 41].

### 4.3 Interpretation

Existing economic evaluations of ovarian cancer testing have examined fewer diagnostic strategies than the present study. For example, Forde et al. [42] compared multivariate index assay and CA125 testing; Kearns et al. [43] compared multimodal screening, ultrasound screening and no screening; Havrilesky et al. [44] looked at annual screening versus no screening.

Westwood et al. [24] assessed various diagnostic tests including RMI, ROMA, and ADNEX risk scores. Their economic evaluation found the ADNEX 10% strategy to be the most cost‐effective option at the £30 000 per QALY threshold in a lifetime Markov model. In contrast, we chose a CCA as a robust approach representing the trade‐offs involved in decision making, given no single outcome measure sufficiently captures patients both with and without ovarian cancer. This approach also allowed us to comprehensively map patient pathways rather than using a simplified version of treatment pathways following an ovarian cancer diagnosis.

## 5 Conclusion

Earlier and better diagnostic strategies are essential to improving ovarian cancer outcomes. This study compared seven of the available diagnostic strategies. A trade‐off involved was found: the usual care strategy, RMI 200, was least costly in both cohorts, but its lower sensitivity resulted in higher missed cancer diagnoses (with poorer survival outcomes) and lower diagnostic yield than an ADNEX 3% strategy. The results suggest a two‐step strategy IOTA ADNEX 10% offers the best balance with near‐comparable expected costs to RMI 200 whilst retaining significantly higher diagnostic yield and lower cancer deaths, making it highly suitable for implementation in practice.

The findings demonstrate that modifications to clinical pathways extend beyond diagnostic test accuracy; they fundamentally influence clinical decision‐making, manifesting in differential costs and consequences across distinct patient cohorts. This is particularly evident in surgical intervention rates, specialist referral patterns, and long‐term patient outcomes including cancer death.

Future economic evaluations should incorporate longer time horizons and account for broader quality‐of‐life impacts to enable further assessment of cost‐effectiveness. Pertinent to this is future research into the quality‐of‐life impact of timely diagnosis, misdiagnosis, and the impact on pre‐menopausal women facing infertility resulting from surgery and chemotherapy.

## Author Contributions

S.S.S., T.E.R., M.M. conceptualized and designed the study. S.J.P., M.M., D.G., E.V.W. and T.E.R. were responsible for the economic analysis: S.J.P. and M.M. did the economic analysis and received advice from D.G., E.V.W. and T.E.R. M.M. supervised the economic analysis. SS contributed to the planning and interpretation of the analysis. F.L.K. and S.W. provided clinical input into the pathways. All authors reviewed the results and the manuscript. J.D., R.A., and K.S. have directly accessed and verified the underlying data reported in the manuscript. All authors had full access to all the data in the study and accept responsibility to submit for publication.

## Ethics Statement

ROCkeTS received ethical approval from NHS West Midlands REC (14/WM/1241) and is registered on the controlled trials website (ISRCTN17160843).

## Consent

Written informed consent was obtained from participants before participation.

## Conflicts of Interest

S.S.S. reports a research grant from AoA Diagnostics for work with samples collected in this study but not reported within this manuscript. S.S.S. reports honoraria from AstraZeneca, Merck, and GSK and consultancy from GSK and Immunogen, all unrelated to this work. S.W. declares consultation fees from Asta Zeneca, Pharma and Abbvie, travel and conference support from Abbvie, all unrelated to this manuscript.

## Supporting information


**Appendix S1:** bjo70074‐sup‐0001‐AppendixS1.docx.

## Data Availability

The data that support the findings of this study are available from the corresponding author upon reasonable request.
